# Synthesizing pseudo-T2w images to recapture missing data in neonatal neuroimaging with applications in rs-fMRI

**DOI:** 10.1016/j.neuroimage.2022.119091

**Published:** 2022-06

**Authors:** Sydney Kaplan, Anders Perrone, Dimitrios Alexopoulos, Jeanette K. Kenley, Deanna M. Barch, Claudia Buss, Jed T. Elison, Alice M. Graham, Jeffrey J. Neil, Thomas G. O'Connor, Jerod M. Rasmussen, Monica D. Rosenberg, Cynthia E. Rogers, Aristeidis Sotiras, Damien A. Fair, Christopher D. Smyser

**Affiliations:** a Department of Neurology, Washington University School of Medicine, St. Louis, MO, United States; b Department of Radiology and Institute for Informatics, Washington University School of Medicine, St. Louis, MO, United States; c Department of Pediatrics, Washington University School of Medicine, St. Louis, MO, United States; d Department of Psychological and Brain Sciences, Washington University School of Medicine, St. Louis, MO, United States; e Department of Psychiatry, Washington University School of Medicine, St. Louis, MO, United States; f Department of Pediatrics and the Masonic Institute for the Developing Brain, Institute of Child Development, University of Minnesota, Minneapolis, MN, United States; g Department of Psychiatry, Oregon Health and Science University, Portland, OR, United States; h Department of Psychiatry, University of Rochester, Rochester, NY, United States; i Department of Pediatrics, University of California Irvine, Irvine, CA, United States; j Department of Psychology, University of Chicago, Chicago, IL, United States; k Department of Medical Psychology, Charité-Universitätsmedizin Berlin, Corporate Member of Freie Universität Berlin and Humboldt-Universität Zu Berlin, Augustenburger Platz 1, 13353, Berlin

**Keywords:** Structural MRI, Synthetic medical images, Deep learning, Multi-atlas fusion, Neuroimaging, Neonate

## Abstract

T1- and T2-weighted (T1w and T2w) images are essential for tissue classification and anatomical localization in Magnetic Resonance Imaging (MRI) analyses. However, these anatomical data can be challenging to acquire in non-sedated neonatal cohorts, which are prone to high amplitude movement and display lower tissue contrast than adults. As a result, one of these modalities may be missing or of such poor quality that they cannot be used for accurate image processing, resulting in subject loss. While recent literature attempts to overcome these issues in adult populations using synthetic imaging approaches, evaluation of the efficacy of these methods in pediatric populations and the impact of these techniques in conventional MR analyses has not been performed. In this work, we present two novel methods to generate pseudo-T2w images: the first is based in deep learning and expands upon previous models to 3D imaging without the requirement of paired data, the second is based in nonlinear multi-atlas registration providing a computationally lightweight alternative. We demonstrate the anatomical accuracy of pseudo-T2w images and their efficacy in existing MR processing pipelines in two independent neonatal cohorts. Critically, we show that implementing these pseudo-T2w methods in resting-state functional MRI analyses produces virtually identical functional connectivity results when compared to those resulting from T2w images, confirming their utility in infant MRI studies for salvaging otherwise lost subject data.

## 1 Introduction

Neonatal and infant neuroimaging is growing in popularity and rapidly expanding its utility in characterizing typical and atypical brain development (Smyser and Neil, 2015; Grayson and Fair, 2017; Graham et al., 2021). Across these investigations, high-quality T1- and T2-weighted (T1w and T2w) structural data have proven critical for generating the accurate segmentations necessary for attaining robust volumetric and surface-based measures, as well as precise localization and mapping of functional magnetic resonance imaging (fMRI) data (Hüppi et al., 1998; Mahapatra et al., 2012; Dubois et al., 2014; Savalia et al., 2017; Reuter et al., 2015). However, non-sedated imaging sessions in neonates are frequently limited in duration, interrupted by arousals, and susceptible to large amplitude movements. Subsequently, these critical structural data are periodically either corrupted or not acquired (Barkovich et al., 2019; Malamateniou et al., 2013). Often, the only substitute for obtaining these required data is rescheduling and re-scanning, which is costly and challenging due to the rapid pace of maturation during this stage of development. These burdens often result in high rates of participant loss for population-based studies.

Recent advances in synthetic medical imaging afford a ready solution to recapture missing structural scans with methods typically falling into one of two categories: deep learning or registration-based. Deep learning methods use relatively large datasets of paired images to learn a non-linear mapping of voxel-to-voxel intensities and synthesize one imaging modality from another. Various iterations of convolutional neural networks (CNNs) in the form of U-Nets (Ronneberger et al., 2015) and generative adversarial networks (GANs) (Goodfellow et al., 2014) have proven effective in cross-modality image estimation. These methods are commonly applied in the generation of synthetic positron emission tomography (PET) (Li et al., 2014; Pan et al., 2018, 2019; Lin et al., 2021) and computed tomography (CT) (Nie et al., 2017; Zhao et al., 2018; Zhang et al., 2018) images from MRI data. Recently, several studies have explored the utility of these networks in longitudinal MRI prediction (Xia et al., 2019; Ravi et al., 2019) and T1w-to-T2w image translation (Dar et al., 2019; Welander et al., 2018), however, methods in these studies have been limited to 2-dimensional (2D) image estimations due to computational complexity. Importantly, the latest research on deep generative methods in MRI suggest that 3-dimensional (3D) models are computationally tractable and have successfully demonstrated T1w-to-fMRI translation in adults (Abramian and Eklund, 2019), as well as predicting future MRI from infant scans (Peng et al., 2020), however, these 3D methods have not yet been applied in neonatal populations nor in the context of T1w-to-T2w translation.

In contrast, registration-based methods offer a solution that does not require a large training dataset, but instead only a small “bank" of template subjects. This method operates by registering images from templates to an individual subject image of interest. The registrations are then applied to the target modality of the template subjects. The registered images from the target modality of the template subjects are then combined based on morphological similarity to create a synthetic version of the subject's target modality. This method has previously been explored in the synthesis of CT maps for PET attenuation correction from MRI data (Burgos et al., 2014; Schreibmann et al., 2010). Advances in nonlinear registration algorithms (Klein et al., 2009; Avants et al., 2009; Sotiras et al., 2013; Ou et al., 2011) and joint fusion (Wang et al., 2013; Artaechevarria et al., 2009, Avants, 2009) have greatly improved image-to-image mapping in brain MRI. As a result, this method has become a popular tool for generating MRI segmentations. However, despite these advances, the utility of this class of algorithms has not yet been explored in the context of T1w-to-T2w translation.

While existing studies using these approaches have predominantly included adult participants, infant and pediatric populations may benefit most from application of these techniques to address the challenges inherent to studying this age group (Barkovich et al., 2019). Based on the successful application of 3D GANs for MR image synthesis (Zhang et al., 2018; Abramian and Eklund, 2019) and pediatric image prediction (Peng et al., 2020), as well as the generation of neonatal atlases using multi-template registration in Alexander et al. (2017), we hypothesized that these two image synthesis approaches could be successfully implemented to perform neonatal T1w-to-T2w translation. In this work, we explore the application of both methods through development of two innovative approaches for synthesizing T2w images from T1w images in neonates. We chose to synthesize images in this direction because ongoing white matter myelination during this critical developmental window results in an inversion of tissue contrast in neonates (Dubois et al., 2014) resulting in T2w images demonstrating higher contrast between cerebral tissue types (Gui et al., 2012), a consideration vital for reliable automated MR image processing (Hüppi et al., 1998; Mahapatra, 2012; Dubois et al., 2014; Savalia et al., 2017; Reuter et al., 2015). First, we extend the 2D CycleGAN proposed by Zhu et al. (2017) and validated by Welander et al. (2018) to 3D volumetric images without the restrictions of paired training data nor the stitching together of 2D slices following image synthesis, heretoafter referred to as “3DGAN-T2w”. Additionally, we propose a registration-based method for synthesis that utilizes state-of-the-art symmetric diffeomorphic image registration (Avants et al., 2009) to calculate highly accurate nonlinear transformations and joint fusion (Wang et al., 2013) to perform image synthesis using a training dataset of paired image sets, heretoafter referred to as “Kaplan-T2w”. We then demonstrate the efficacy of these image translation methods in two independent neonatal cohorts. Finally, we establish the efficacy of utilizing synthetic images for application of resting-state functional MRI (rs-fMRI) processing by demonstrating connectivity estimates are highly comparable between data processed with original and synthetic T2w data.

## 2 Methods

### 2.1 Samples

#### 2.1.1 Early life adversity biological embedding (eLABE)

MRI data from 127 neonates (postmenstrual age=41.1 ± 1.5 weeks, female *N* = 59, white *N* = 42) with high-quality (i.e., little to no motion) T1w and T2w images participating in the early life adversity and biological embedding study were used in this analysis. Of the 127 total neonates, MR data from 107 neonates were used as reference and training data for the pseudo-T2w generation methods (see Supplemental Information (SI) “3D-CycleGAN Additional Analyses” for training data quantity comparison), and 20 neonates were used for primary analyses. This study was approved by the Washington University Human Studies Committees and informed consent was obtained from the parents of all participants.

Participants were scanned within the first month of life during natural sleep without the use of sedating medications on a Siemens 3T Prisma scanner with a 64-channel head coil. T1w (TR=2400 ms, TE=2.22 ms, 0.8 mm isotropic), T2w (TR=4500 ms, TE=563 ms, 0.8 mm isotropic), spin echo fieldmaps (SEFM) (TR= 8000 ms, TE=66 ms, 2 mm isotropic, MB=1), and rs-fMRI data (TR=800 ms, TE=37 ms, 2 mm isotropic, MB=8) were collected. rs-fMRI data were collected in both anterior → posterior (AP) and posterior → anterior (PA) phase encoding directions. Each BOLD run consisted of 420 frames (5.6 min), with a minimum of 2 runs (11.2 min) and maximum of 7 runs (39.2 min) collected per scanning session.

#### 2.1.2 Environmental influences on child health outcomes (ECHO)

The ECHO Program is a nationwide study conducting observational studies of pediatric cohorts including participants of different races, genders, ages, and backgrounds to better understand the effects of environmental influences on child health and development. Informed consent was obtained from the parents of all participants.

MRI data from 10 infants (age=41.2 ± 1.9 weeks, female *N* = 5, white *N* = 8) with high-quality (i.e., little/no motion) T1w and T2w images acquired at the University of Pittsburgh as part of the ECHO Study were included in supplemental analyses as a replication cohort. These participants were scanned during natural sleep without the use of sedating medications on a 3T Siemens Prisma scanner with a 64-channel head coil. The following sequences were acquired for each participant: T1w (TR=2400 ms, TE=2.22 ms, 0.8 mm isotropic), T2w (TR=3200 ms, TE=563 ms, 0.8 mm isotropic), and up to four 5 min rs-fMRI scans (TR=800 ms, TE=37 ms, 2 mm isotropic, MB=8). rs-fMRI data were collected in the AP phase encoding direction only.

### 2.2 Data analysis

#### 2.2.1 Pseudo-T2w method 1: 3DGAN-T2w

**Fig. 1:**
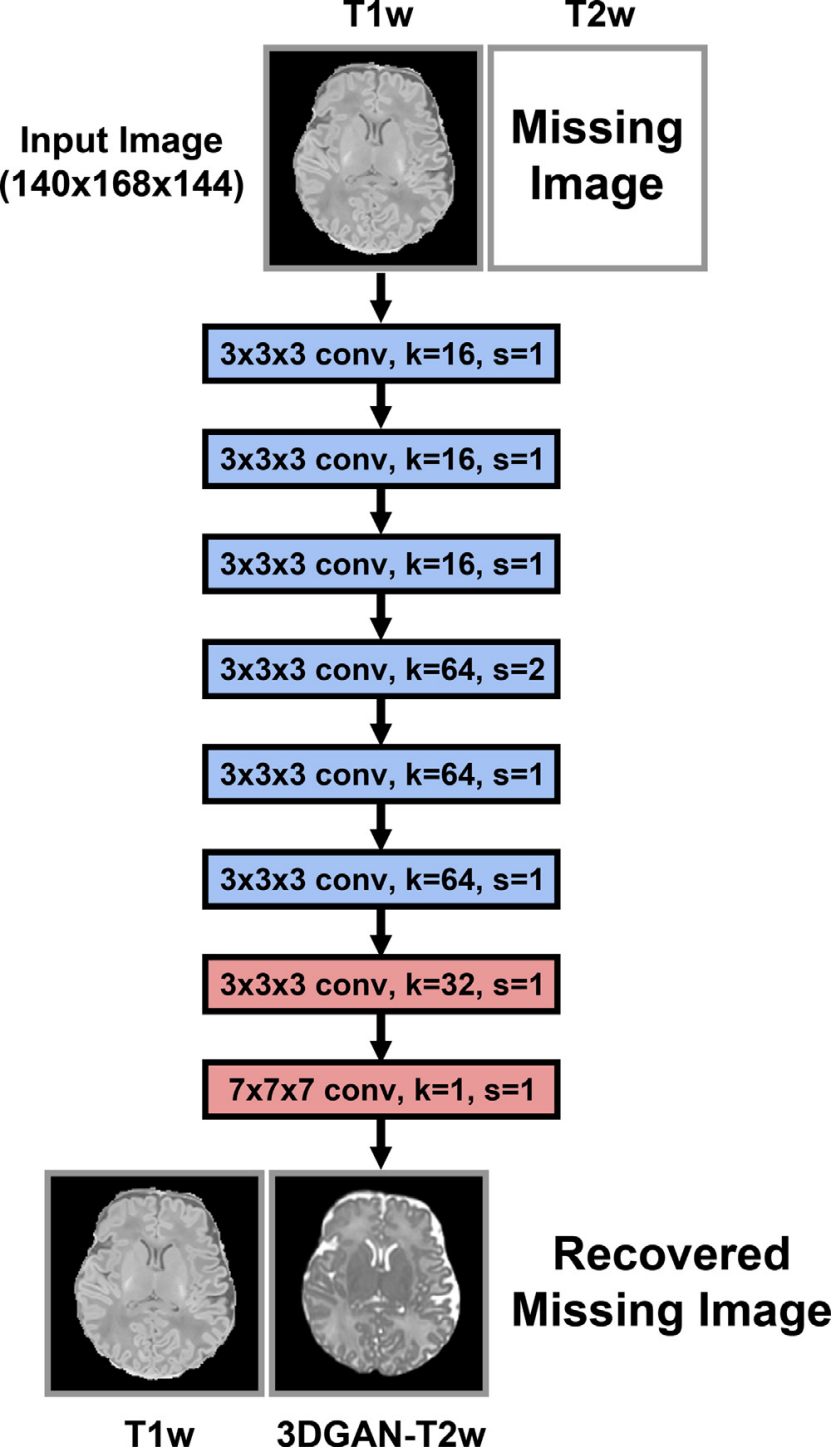
*3DGAN-T2w Generation Network Architecture*. Missing T2w images can be generated directly by simply inputting the full volumetric T1w image into this network. The generator consists of an “encoding stage” (blue) and a “decoding state” (green). k refers to the number of kernels and s refers to the stride of the convolutions at each layer. The encoding stage is made up of 6 3D convolutional layers that take the full resolution input (140 × 168 × 144) and output a latent representation that has been downsampled by half after the fourth layer. Each convolutional layer is followed by a ReLU activation. The decoding stage upsamples the latent representation back to the size of the original input using 2 transpose convolutions, and finally estimates the T2w using convolution with a 1 × 1 × 1 kernel and a hyperbolic tangent (“tanh”) activation function. This network was trained using the CycleGAN procedure outlined in the SI section “3D-CycleGAN Additional Analyses”.

The deep learning model for creating a 3DGAN-T2w consists of multiple CNNs trained simultaneously with the goal of learning a non-linear mapping between T1w and T2w images. The networks consist of two image generators and two image discriminators. One of the generators attempts to estimate T2w images from T1w images and is depicted in Fig. 1, while the corresponding discriminator distinguishes real from synthesized T2w images. Similarly, the remaining networks are trained with the goal of creating pseudo-T1w from T2w images. The networks are trained simultaneously using two separate loss functions: adversarial loss and cycle-consistency loss. In adversarial loss, the discriminator attempts to classify the resulting pseudo-T2w and real images, and the weights are updated based on the mean squared error between the discriminator's prediction and true label of the image. This loss is minimized for the discriminator network to improve the ability to detect synthetic images, but the loss is maximized for the generator network to create more realistic synthetic images. As one network's performance improves, the other must necessarily improve as well. In cycle-consistency loss, the pseudo-T1w generator attempts to estimate the T1w from the resultant pseudo-T2w. In theory, the pseudo-T1w image should be identical to the original T1w image, so the mean absolute error between these two is used to further update the generator. The full CycleGAN training architecture is depicted in Supplementary Fig. 1.

The original 2D model on which this architecture was based, while useful and efficient in comparing different types of GANs, yields banding artifacts in 3D medical images since synthetic images are generated slice-by-slice. To resolve this issue, the model was extended to 3D so that it can be trained on full volumetric data. This extra dimension comes at the cost of substantially increasing the memory requirements of the network and therefore must be trained using a GPU with at least 32 GB of VRAM. To accommodate training, it was necessary to remove some of the deeper layers as well as the number of filters at each layer, with training finishing in roughly one week. The generator network in this work was trained using paired T1w and T2w images from 107 eLABE neonates and is visualized in Fig. 1.

#### 2.2.2 Pseudo-T2w method 2: Kaplan-T2w

Generating a Kaplan-T2w requires a set of high-quality reference data, which includes aligned T1w and T2w images. Reference data were separated into age-specific “banks”, where each “bank” consisted of 10 subjects that were scanned within 2–3 weeks postmenstrual age (PMA) of each other to account for differences in the rapidly developing neonatal brain.

A Kaplan-T2w image is generated to anatomically match an individual subject T1w image. In order to maximize anatomical correspondence between the generated Kaplan-T2w and the T1w image, computations involved in creating the Kaplan-T2w were restricted to voxels within the brain by applying a manually drawn brain mask so that regions of non-interest (i.e., the body and surrounding air) did not contribute to similarity optimization. Additionally, all images were bias field corrected using the ANTs software package in an effort to remove inhomogeneities (Avants et al., 2009; Tustison et al., 2010).

**Fig. 2:**
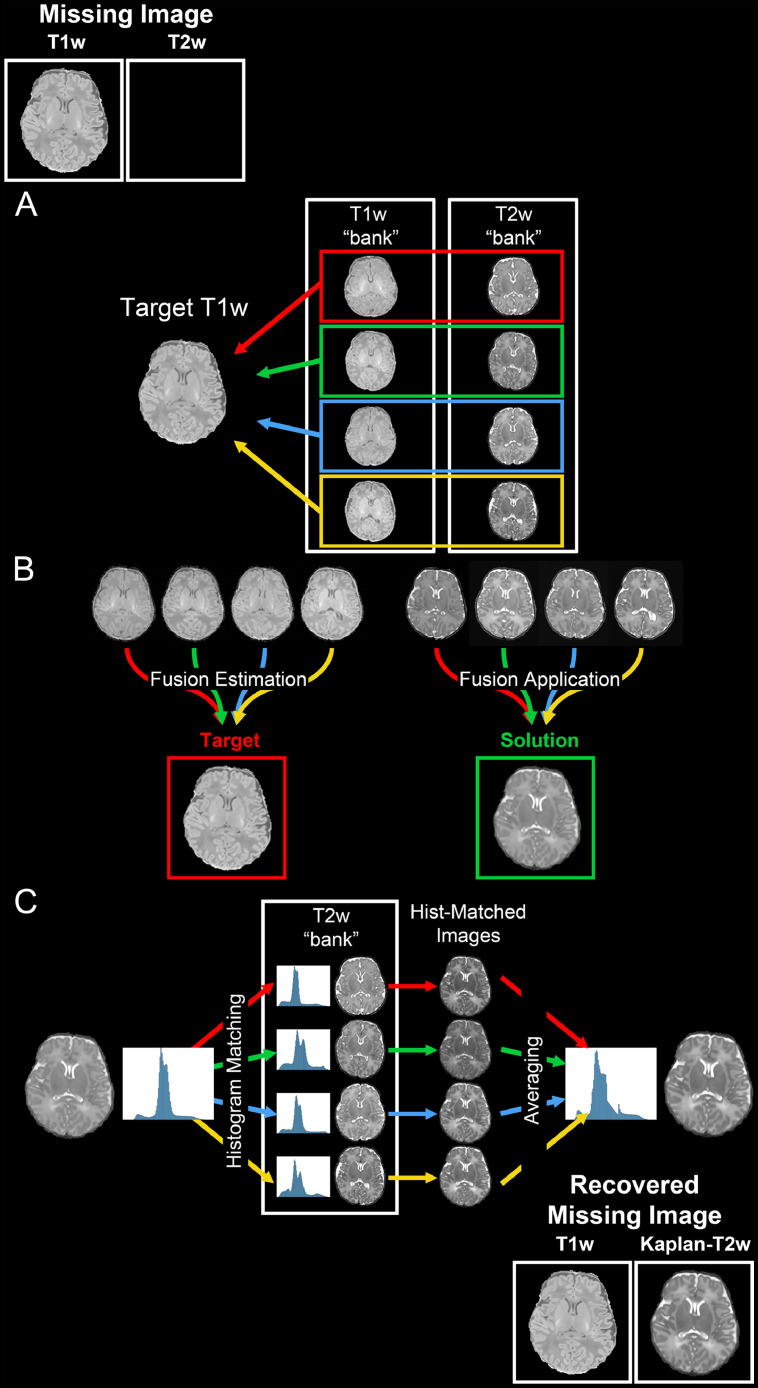
*Kaplan-T2w Generation Procedure*. Missing T2w images can be recovered by generating a Kaplan-T2w from a T1w image. The procedure to do so begins with (A) nonlinear registration between each T1w “bank” image and the target T1w image. The estimated deformation is then applied to the T2w and segmentation “bank” images, resulting in 3 registered images for each “bank” subject. (B) The optimal fusion weighting of the registered “bank” images to the target T1w is estimated using only the registered T1w “bank” images. The computed fusion weighting is then applied to the registered T2w and segmentation “bank” images resulting in fused images that are structurally comparable to the T1w target image. (C) Contrast, texture, and quality of the fused T2w image is improved by performing a histogram matching to all “bank” T2w images. These are then averaged to create the final Kaplan-T2w image.

With the intention of directly mapping each “bank” image to the target individual, ANTs registration tools were used to estimate the deformation field between each of the “bank” T1w images to the target individual T1w image. Applying the calculated nonlinear transformations to each “bank” T1w and T2w image produced 10 estimations of the target for each modality (Fig. 2a). Given that multi-atlas fusion techniques produce superior representations of a target image in comparison to any single estimation alone (Rohlfing et al., 2004; Heckemann et al., 2006), ANTs Joint Fusion was used to determine the optimal fusion weighting of the 10 T1w estimates that best represent the individual target T1w (Fig. 2b). These weights were then applied to the set of T2w estimates, resulting in a pseudo-T2w image that is structurally accurate to the individual target T1w. In order to improve the texture of the pseudo-T2w image, ANTs DenoiseImage was applied. Since histogram manipulation has been shown to improve image contrast and quality (Senthilkumaran and Thimmiaraja, 2014; Patel et al., 2020), this image was histogram matched to each of the 10 “bank” T2w images in order to provide realistic image improvements. This resulted in 10 pseudo-T2w estimations; these were then averaged to produce a realistic pseudo-T2w brain, depicted in Fig. 2c. The skull and surrounding background noise were mapped similarly to the individual target and added to the brain-only image resulting in the final Kaplan-T2w image. The entire process was completed utilizing multiple CPUs in a matter of hours.

#### 2.2.3 Structural and functional data processing

T1w and T2w MR images were corrected for gradient and readout distortions using the methods described in (Glasser et al., 2013), and distortion corrected images were denoised using ANTs DenoiseImage (Avants et al., 2009; Manjón et al., 2010). Anatomical segmentations and surfaces were generated using MCRIBS (Adamson et al., 2020), where either a T2w image or pseudo-T2w image was used as input. All segmentations were manually inspected and corrected as needed by experienced raters (DA, JD, DM). rs-fMRI data were preprocessed through a standard neonatal BOLD preprocessing pipeline using a combination of the 4dfp tool suite (ftp://imaging.wustl.edu/pub/raichlab/4dfp_tools/; Shulman et al., 2010) and FSL tools (Jenkinson et al., 2012). BOLD timeseries data were corrected for intensity differences due to interleaved acquisition and debanded. Rigid body motion within BOLD runs was corrected using linear realignment. Images were bias field corrected and normalized to whole brain mode 1000. Time series data were corrected for readout distortion and linearly registered to 711–2N Talairach atlas space (Smyser et al., 2010) as: BOLD→individual T2w or pseudo-T2w→cohort-specific T2w atlas→711–2N Talairach atlas, with linear registrations performed in a single step. The cohort-specific T2w atlas was generated using ANTs atlas builder from 50 eLABE subjects that were independent of the 20 test subjects used in analyses. Atlas registered BOLD timeseries were mapped to subject-specific surfaces using methods adapted from Marcus et al. (2013) and Marcus et al. (2011). Frame censoring was performed so that only data with at least three consecutive frames with frame displacement (FD) < 0.25 mm were used. Each BOLD run was demeaned, detrended, and regressed for nuisance waveforms including: white matter, ventricular and extra-axial cerebrospinal fluid (CSF), whole brain, and the 24-Friston motion parameters. Data were then bandpass filtered (0.005–0.1 Hz) to remove non-BOLD frequencies and spatially smoothed.

#### 2.2.4 Evaluation of structural data

To evaluate the accuracy of the generated pseudo-T2w images, they were compared to the corresponding ground-truth T2w images by computing the absolute value of the intensity differences between them. Images were first normalized to the same scale (0–2000) and the absolute error was restricted to voxels that fell within the brain. To summarize the performance for each individual, the mean of the absolute errors (MAE) across all voxels within the brain was computed.

To assess the correctness of anatomical structure of the pseudo-T2w images, the mean structural similarity index (MSSIM) was computed between each pseudo-T2w and T2w image (Wang et al., 2004). The images were first normalized to the same scale (0–2000) and the calculation was restricted to voxels that fall within the brain. To validate the structural similarity of the MCRIBS output (Adamson et al., 2020), the DICE coefficient was computed between the atlas registered cortical ribbons derived from the psuedo-T2w and T2w images.

To gauge the contrast properties important for accurate segmentation and registration, the contrast-to-noise ratio (CNR) of the anatomical images was computed. Here, CNR is defined as

(1) $$CNR=\frac{\left|\mu \left(GM\right)-\mu \left(WM\right)\right|}{\sqrt{(\sigma {\left(GM\right)}^{2}+\sigma {\left(WM\right)}^{2})/2}}$$

where *µ(GM)* and *σ(GM)* are the average and standard deviation of all voxels within the gray matter region-of-interest (ROI), and *µ(WM)* and *σ(WM)* are the average and standard deviation of all voxels within the white matter ROI (Lee and Riederer, 1987). MCRIBS (Adamson et al., 2020) anatomical segmentations generated from the T2w or pseudo-T2w images were adapted to obtain gray and white matter ROIs that minimize partial volume averaging. To construct the gray matter ROI, the gray matter segmentation was shifted inward by both 1 and 2 voxels, and the 2-voxel shift mask was then removed from the 1-voxel shift mask so that only the center of the segmentation remained. White matter ROIs were generated by eroding the white matter segmentations by 5 voxels.

CNR was computed to confirm that pseudo-T2w images possess similar contrast compared to T2w images. Paired t-tests were then performed between all combinations of anatomical image types to determine the significance of differences in CNR, using a threshold of *p* < 0.05 to denote significance.

#### 2.2.5 Evaluation of synthetic images in fMRI analyses

The 4dfp tool suite (ftp://imaging.wustl.edu/pub/raichlab/4dfp_tools/; Shulman et al., 2010) was used to compute linear registrations between BOLD and anatomical data (T2w and pseudo-T2w images) to 711–2N Talairach atlas space. Registrations optimized the gradient correlation between images and were computed BOLD → individual anatomic image → cohort-specific T2w atlas → 711–2N Talairach atlas. To assess the quality of BOLD to anatomical and anatomical to atlas registrations, the mutual information (MI) between each registered image and its target was computed (Avants et al., 2009). MI measures the amount of shared information between two images and has the ability to capture nonlinear relationships in image intensities (Viola and Wells, 1995; Collignon et al., 1995). This property is ideal for evaluating registrations of multi-modality images, which often have nonlinear relationships. Paired *t*-tests were then performed between the computed MI for all combinations of anatomical images to determine the significance of differences in registration quality, using a threshold of *p* < 0.05 to denote significance. See SI “Additional Comparisons” for further analysis comparing the registration quality to the T1w.

To assess brain-wide similarities of BOLD data that were pre-processed using either a T2w or pseudo-T2w image, functional dense connectomes (dconns) from each participant's rs-fMRI data were computed for each of the three anatomic images. An average dconn for each of the three pre-processing methods, T2w, 3DGAN-T2w, and Kaplan-T2w, was obtained by averaging across participants. Pearson correlation coefficients were computed between the T2w average dconn and each of the pseudo-T2w average dconns to measure similarity.

Functional connectivity (FC) estimates for BOLD data pre-processed using T2w and pseudo-T2w images were obtained by computing the pairwise correlation of the average BOLD time series for a set of standard cortical parcels (Gordon et al., 2016). Matrices consisting of these FC estimates for each participant were then organized based upon age-specific resting state network assignments (RSN) determined using previously published methods (Wheelock et al., 2019; Eggebrecht et al., 2017). The average and variance of the connectivity matrices were computed across subjects to evaluate similarity in RSN connectivity patterns between BOLD data pre-processed using T2w and pseudo-T2w images. Paired t-tests between the T2w connectivity matrices and both of the pseudo-T2w connectivity matrices were then performed to determine the significance of differences in FC estimates, using a Bonferroni corrected threshold of *p*<0.00015 to denote significance. Bonferroni correction was computed as

(2) $$p<\frac{0.05}{N_{parc}}$$

where *N_parc_* = 333 parcels.

## 3 Results

### 3.1 Anatomical comparison of T2w and pseudo-T2w images

**Fig. 3:**
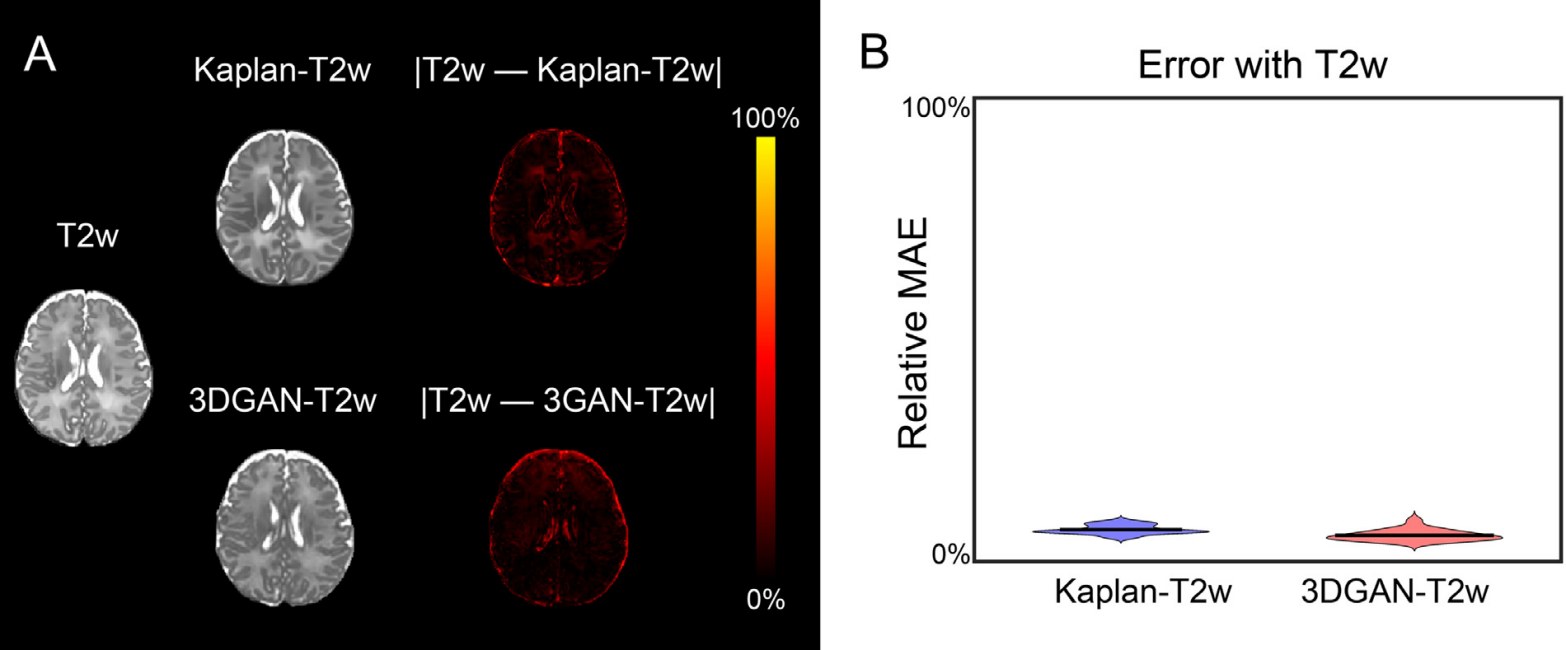
*Error in pseudo-T2w images.* (A) Heatmap of absolute error between each pseudo-T2w image and the corresponding T2w image for a representative subject, where 100% represents the highest error value between the images. Here, brighter values indicate larger error and are localized to regions of CSF. (B) Violin plot depicting the relative MAE with T2w images of all subjects for both peudo-T2w images (Kaplan-T2w 6.9 ± 0.9%, 3DGAN-T2w 5.6 ± 1.1%). Smaller values indicate less error.

Resulting absolute error for a representative subject is presented in Fig. 3a where brighter voxels represent the largest error. Here, the overall error in both pseudo-T2w images is low and localized to the CSF. The relative mean absolute error (MAE) of included voxels for all subjects is presented in Fig. 3b. Relative MAE across subjects was 6.9 ± 0.9% for the Kaplan-T2w images and 5.6 ± 1.1% for the 3DGAN-T2w images.

**Fig. 4:**
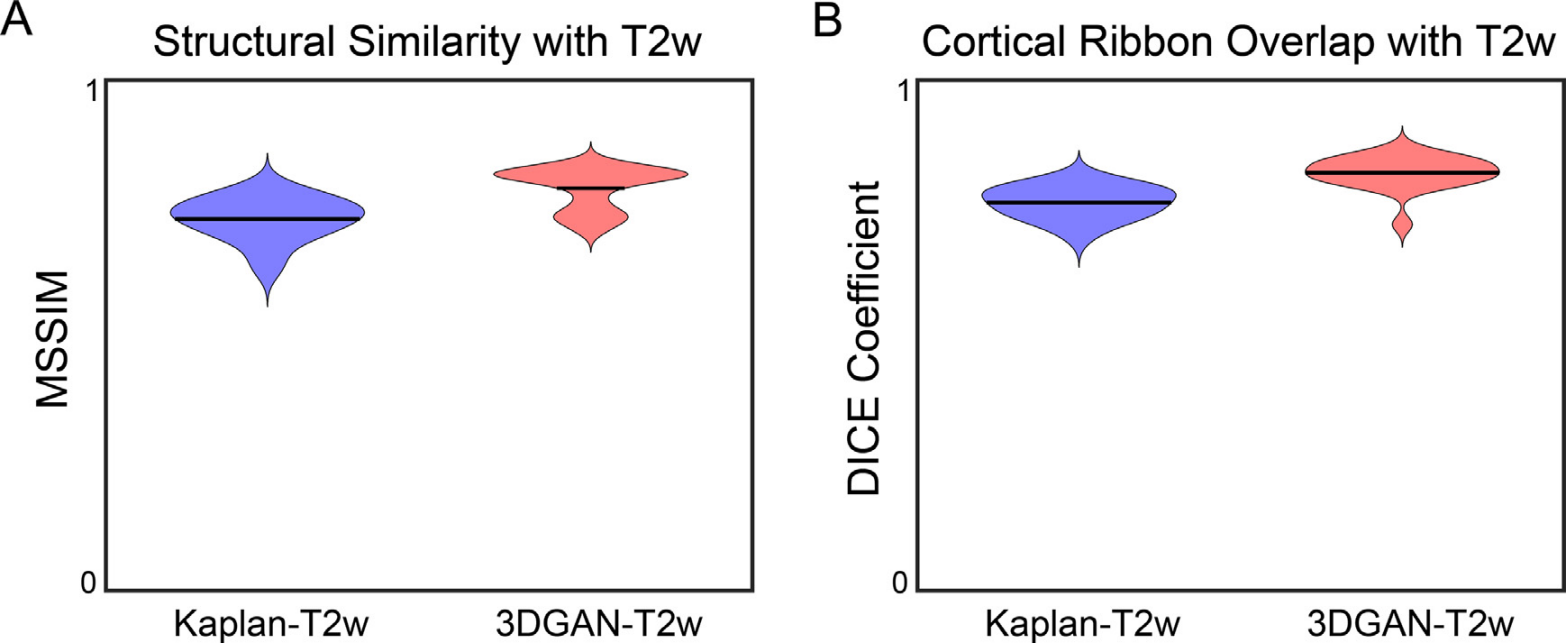
*Structural comparison of pseudo-T2w images to T2w images*. Violin plots depicting the (A) MSSIM of the images and (B) DICE coefficient of the cortical ribbons between the T2w and each psuedo-T2w for all subjects. For both metrics, values closer to 1 indicate higher similarity.

Anatomical similarity between pseudo-T2w and T2w images across subjects is presented in Fig. 4. MSSIM (Fig. 4a) was 0.72±0.04 for the Kaplan-T2w images and 0.79±0.04 for the 3DGAN-T2w images, and cortical ribbon DICE coefficients (Fig. 4b) were 0.76±0.03 for the Kaplan-T2w images and 0.82±0.03 for the 3DGAN-T2w images. See SI “Additional Comparison” for visual comparison of pseudo-T2w and T2w cortical ribbons.

**Fig. 5:**
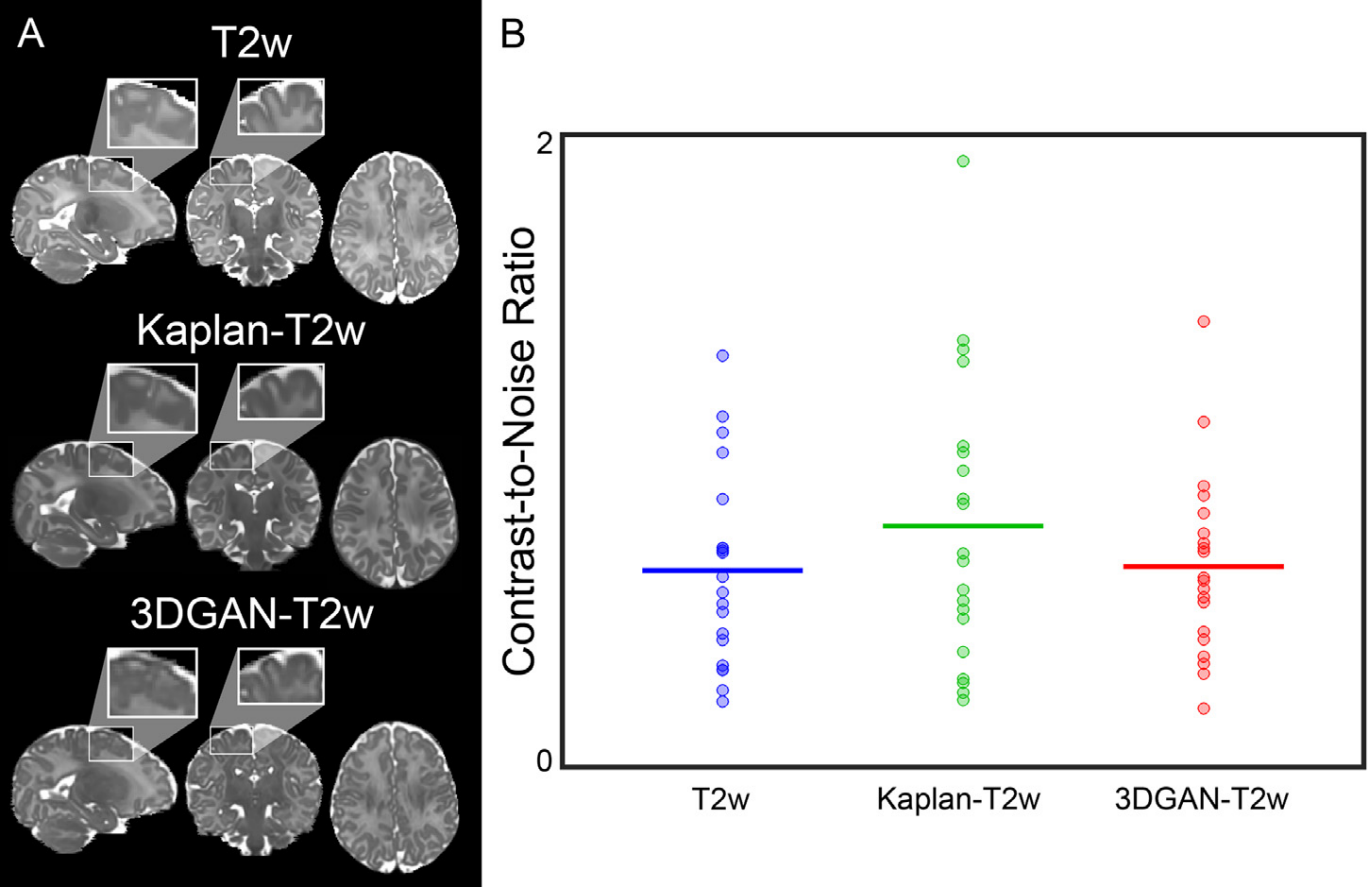
*Contrast comparison of anatomical images*. (A) Volumetric image slices from T2w and pseudo-T2w images for a representative subject. Cropped regions highlight contrast properties of each image between gray and white matter. Visual contrast properties are quantified as the (B) CNR between gray and white matter of different anatomical image types. CNR distributions are equivalent between T2w (0.62±0.31), Kaplan-T2w (0.76±0.45), and 3DGAN-T2w (0.63±0.29) images.

Volumetric slices of anatomic images from a representative participant are presented in Fig. 5a. Fig. 5a demonstrates the qualitative similarities in contrast properties between the pseudo-T2w and T2w images that are important for segmenting tissues, as well as computing optimal registrations between images automatically. These qualitative observations of tissue contrast can be quantitatively measured using CNR, depicted in Fig. 5b for T2w and pseudo-T2w images, where increased CNR corresponds to improved contrast necessary for subsequent processing. Plotted mean lines show no significant CNR differences between T2w images and both pseudo-T2w images (T2w with: Kaplan-T2w *p* = 0.15, 3DGAN-T2w *p* = 0.71).

### 3.2 fMRI pre-processing with pseudo-T2w images is comparable to T2w images

**Fig. 6:**
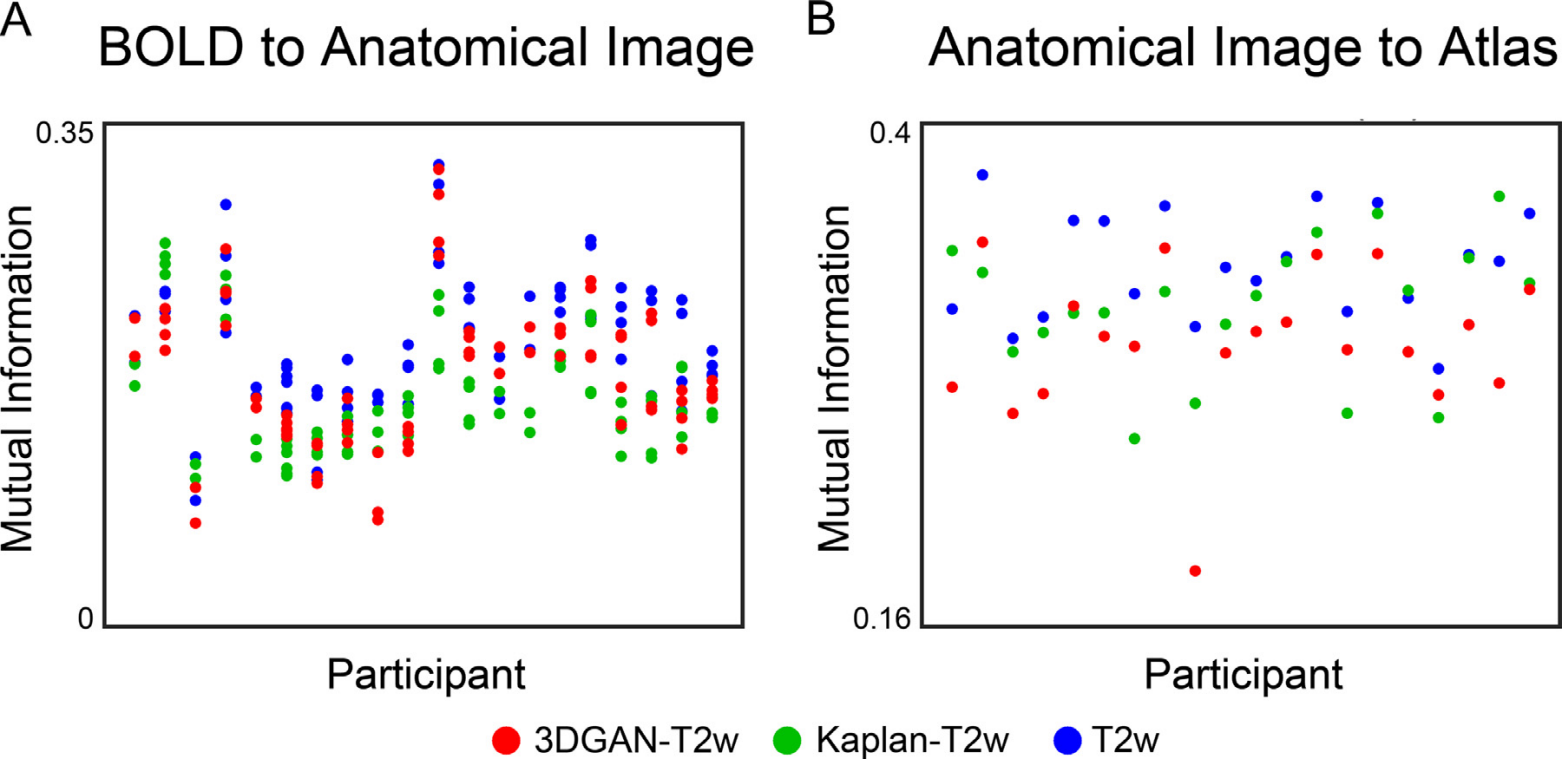
*Registration accuracy of BOLD and anatomical images.* Mutual information (MI) for each participant between registered (A) BOLD and anatomical images, as well as registered (B) anatomical and atlas images for each anatomical image type. Note the overlap in MI between T2w and pseudo-T2w images for both BOLD and anatomic image registrations.

Since registration algorithms typically rely on intensity differences between tissues to perform alignment (Gonzalez-Castillo et al., 2013), greater tissue contrast translates to better registration between images. Fig. 6 shows the quality of registration of BOLD to anatomic data (Fig. 6a), as well as anatomic data to the 711–2N Talairach atlas (Fig. 6b). Here, higher quality registration is defined as increased MI, and there is substantial overlap between the MI of pseudo-T2w and T2w registrations. However, the T2w registration quality was higher than both pseudo-T2w images (BOLD registration: *p* < 0.001 for both Kaplan-T2w and 3DGAN-T2w; anatomical registration: *p* = 0.002 for Kaplan-T2w and *p* < 0.001 for 3DGAN-T2w). Between the pseudo-T2w registrations, registration quality for Kaplan-T2ws was higher than 3DGAN-T2ws for anatomical registrations (*p* = 0.04), but 3DGAN-T2ws were higher for BOLD registration (*p* < 0.001).

**Fig. 7:**
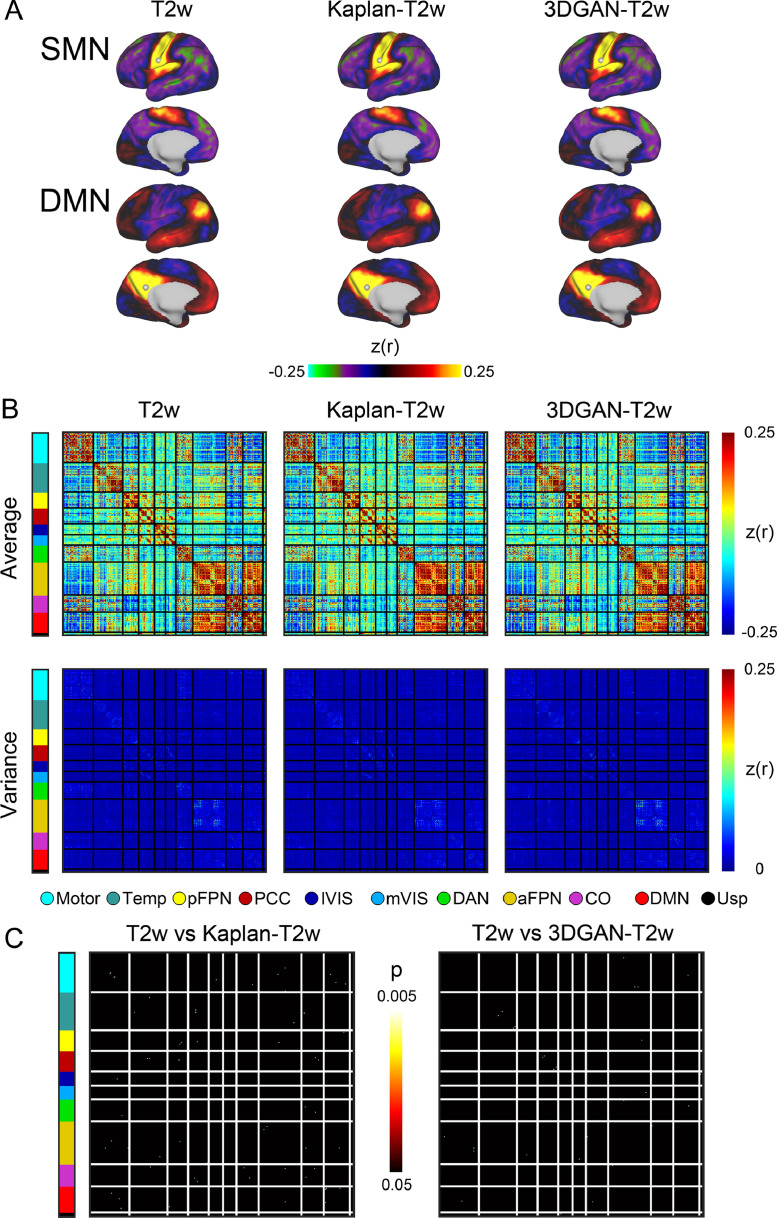
*Pseudo-T2w use in functional connectivity*. (A) Average functional dense connectomes at select seeds for BOLD data pre-processed with T2w and psuedo-T2w images. Seeds were selected as the center vertex of adult-defined network clusters from early developing networks. Seedmaps from the somatomotor (top row) and default mode (bottom row) networks show similar connectivity patterns across the three anatomical image types. (B) Average functional connectivity matrices (top row) using cortical parcels and the variance of the connectivity matrices (bottom row) across participants for BOLD data pre-processed with T2w and pseudo-T2w images. Parcels are organized into networks including: motor, temporal lobe (Temp), posterior frontoparietal (pFPN), posterior cingulate cortex (PCC), lateral visual (lVIS), medial visual (mVIS), dorsal attention (DAN), anterior frontoparietal (aFPN), cingulo-opercular (CO), default mode (DMN), and unassigned (Usp). Note similar patterns and connectivity strength between matrices of T2w and pseudo-T2w images. (C) FC estimate differences between BOLD data pre-processed with T2w images and Kaplan-T2w or 3DGAN-T2w images. Brighter colors indicate more significant differences.

Group average dconns for BOLD data pre-processed using T2w and pseudo-T2w images are shown in Fig. 7a. Select seeds from early developing brain networks are shown, where seeds are taken from the center of adult-defined network clusters (Power et al., 2011). Note the similar connectivity patterns between the seedmaps of the pseudo-T2w and T2w maps. The brain-wide likeness is further reflected when correlating the dconns of pseudo-T2w and T2w maps (*r* = 0.98 for both Kaplan-T2w and 3DGAN-T2w).

In addition to brain-wide similarities, Fig. 7b demonstrates regional connectivity patterns of BOLD data pre-processed with pseudo-T2w and T2w images. The first row displays the average connectivity matrices for all three pre-processing methods. FC patterns are consistent between all three average connectivity matrices, both within and between RSNs. To demonstrate the spread across subjects, the variances of the connectivity matrices are plotted in the second row. Overall, the variance is low for all three pre-processing methods, but notably, regions of higher variance occur in the same parcels regardless of the anatomic pre-processing method.

To confirm equivalency between T2w and pseudo-T2w connectivity matrices, Fig. 7c shows statistical differences measured using a paired *t*-test. Parcels identified as different are sparse with no apparent patterns, nor are they isolated to any particular region. Additionally, overall differences are minimal (0.06% for Kaplan-T2w and 0.03% for 3DGAN-T2w). All analyses and results were replicated in the ECHO cohort, see SI ECHO Cohort Results”.

## 4 Discussion

In this work, we developed two techniques to generate accurate synthetic T2w images from T1w images in neonates. In the first method, we extended prior 2D deep learning models to 3D, avoiding the banding issues associated with 2D models. Alternatively, we proposed a second method that makes use of recent advances in nonlinear registration and builds upon prior work to perform image-to-image translation. Notably, we have shown that T2w and synthetic T2w images are anatomically alike, possess similar contrast properties, and provide accurate targets for BOLD image registration. Further, we demonstrated that pseudo-T2w images produce equivalent results to T2w images for rs-fMRI pre-processing, surface mapping, and connectivity estimates in two independent cohorts. Crucially, implementing these techniques affords the ability effectively recover potentially otherwise lost participant data.

### 4.1 Prior utilization of synthetic image methods

Deep learning continues to allure researchers with promises of an all-powerful model capable of generating multi-contrast images with tunable image acquisition parameters (Denck et al., 2021). While recent developments in the form of GANs have enabled training of such models without the requirement of paired data, sizable datasets with a wide variety of image types are still required. Recent variations of the classic GAN (Goodfellow et al., 2014), including TarGAN (Chen et al., 2021), Hyper-GAN (Yang et al., 2021), and PTNet (Zhang et al., 2021) among others, have reported new improvements to network architecture to optimize cross contrast MR image estimation. However, in an effort to limit computational resources, these models are critically restricted to computations on 2D slices, which has been shown to result in discontinuities and artifacts in the final synthetic volumetric image (Xiang et al., 2018). This major limitation of these deep learning algorithms has been highlighted by researchers attempting to impute missing data for longitudinal studies (Peng et al., 2021). To address this issue, Peng et al. conceded image resolution and downsampled cropped input images by a factor of two. However, because high-resolution images are optimal for anatomical image preprocessing, we instead opted to reduce the parameters of the model to address this limitation. Subsequently, in this study, we demonstrated these shallower CNNs are equally capable of generating 3D volumes that can be successfully implemented in current research applications. Additionally, while 3D cycleGANs have previously been implemented for various medical image synthesis tasks in adult populations (Zhang et al., 2018; Abramian and Eklund, 2019; Pan et al., 2018, 2019), to the best of our knowledge this is the first to do so in the context of neonatal T1w-to-T2w applications.

Alternatively, registration based methods have been thoroughly investigated and remain the gold standard for brain segmentation even with the availability of deep learning methods (Iglesias and Sabuncu, 2015), though their use in image translation has been predominantly overlooked in recent literature. Earlier attempts by Schreibmann et al. (2010) to synthetically translate MR images to CT utilized deformable registration; however, their approach only included a single atlas, which has been shown to underperform compared to multi-atlas fusion (Rohlfing et al., 2004; Heckemann et al., 2006). While multi-atlas fusion techniques typically use template image intensity to inform fusion weightings for segmentation (Wang et al., 2013), the same methodology has not been fully explored to create synthetic images. Although in prior work (Burgos et al. 2014) utilized multi-atlas fusion for PET attenuation correction, their method did not exploit recent advances in diffeomorphic registration which has proven advantageous for high variability in deformation magnitudes, a common issue in the developing brain (Klein et al., 2009; Avants et al., 2009; Rogelj and Kovacic 2006; Trouvé 1998; Beg et al., 2005). Further still, previous work has not addressed the anatomical accuracy of translated images to the degree found in highly detailed MR images. In this work, we build upon these efforts and used image intensity fusion and optimal nonlinear registration to demonstrate that multi-atlas fusion is capable of generating accurate anatomical images in normative populations.

### 4.2 Comparison of pseudo-T2w method requirements

Overall, both pseudo-T2w methods produce highly accurate anatomical images that can be used interchangeably with T2w images for rs-fMRI analyses. Despite minor differences in performance for metrics evaluated in this work, the two pseudo-T2ws primarily differ in their methodological requirements, including data availability, as well as system resources and computational time. While both pseudo-T2w methods require high-quality (i.e., little to no motion) reference images, the quantity of reference data differs greatly between the two: roughly 10 subjects for Kaplan-T2w compared to about 70 subjects for the initial training of the 3DGAN-T2w. This becomes particularly important for studies with limited data, as it leaves the Kaplan-T2w method as the primary option for developing a new model. However, if a pre-trained 3DGAN-T2w model exists, lower quantities of new data could be incorporated by further training the model.

Because the initial training of the 3DGAN-T2w method requires large quantities of data, it must be run on a GPU with at least 32 GB of VRAM and takes approximately one week to train the model. However, once the network training is complete, 3DGAN-T2w images can be produced within minutes on a CPU. In contrast, the Kaplan-T2w method generates images within a couple hours and is executed on CPUs. Since CPUs are more cost effective and widely available, producing Kaplan-T2w images might be more readily implementable. Importantly, both methods utilize software that is publicly available on most operating systems.

The procedures behind the pseudo-T2w methods also lead to differences in their potential ability to incorporate new sequence parameters. For instance, it is straightforward to add new sequences to an existing pre-trained 3DGAN-T2w model for subsequent training and refinement without the need for paired data. In contrast, the Kaplan-T2w method would require a new set of paired data to accommodate substantially different sequences. As was shown in the ECHO dataset results, while generated pseudo-T2w contrast properties are specific to the sequences in the training data, both methods generalize well to new sequence parameters in terms of resultant anatomical accuracy and subsequently derived measures. Potential considerations for training a new model include: age of cohort, tolerance of contrast deviation, and input image variation. First, since T1w and T2w contrast properties flip during the first few months of life (Dubois et al., 2014), it would be necessary to train a new model for each period of development. In addition, output image contrast properties cannot be extrapolated outside of the training set, so the need to train a new model depends on the tolerance of deviation in the training set's contrast properties from that of a particular study. Finally, a new model would need to be trained if the input images differ substantially from the training set, and further investigation into acceptable input image parameter bounds is needed. Ultimately, both methods are sufficiently well-suited for MR analyses, and pseudo-T2w method selection should be determined by the needs and resources of a particular study.

### 4.3 Implementation of synthetic images in MR analyses

Given that most MR analyses rely on T1w and T2w images for determining spatial and structural information, anatomical accuracy in synthetic images is essential. Even moderate errors in anatomy can result in incorrect volumetric measures or improper localization of functional activity, which can bias study conclusions. We have demonstrated that the two methods for synthesizing pseudo-T2w images presented in this work are appropriate for use in MR analyses due to their high anatomic accuracy when compared to corresponding T2w images. Critically, implementation of both methods permits the use of any processing stream regardless of which anatomical image is collected.

In rs-fMRI analyses, anatomical images typically serve as intermediate targets for BOLD registration to atlas templates and are used to generate surfaces and tissue segmentations for delineating nuisance signals. Therefore, it is crucial for BOLD data to precisely align with anatomical images to ensure nuisance signals are correctly demarcated and for accurate anatomical atlas registration to compare rs-fMRI data across individuals. Tissue contrast has been shown to greatly affect the quality of image registration estimates (Gonzalez-Castillo et al., 2013). Herein, we have shown that, for neonates, T2w and pseudo-T2w images have similar tissue contrast that, more importantly, translates to the precise registration necessary for correct surface mapping and nuisance regression. Between the pseudo-T2w methods, minor differences in registration performance may be attributed to the higher CNR for the Kaplan-T2w and lower MAE for the 3DGAN-T2w; but more importantly, both methods result in highly accurate registrations overall. Once the cortical signal is accurately identified, cortical brain-behavior outcomes are generally investigated regionally at the parcel or network level. To best assess the effect that pre-processing BOLD data with pseudo-T2w images has on a typical study of FC, we computed FC estimates using a set of standard cortical parcels (Gordon et al., 2016). By showing that there is no effective difference between both pseudo-T2w and T2w FC estimates, we demonstrate that implementing pseudo-T2w fc processing likely will not impact study outcomes in this age range and can therefore be confidently used when T2w images are not available.

### 4.4 Limitations and future work

This work was limited to data obtained from healthy term-born neonates. Further investigation is needed to determine the applicability of these methods to premature, injured, or other atypically developed brains, as well as different age groups. Further, although network-level fc estimates were evaluated using networks derived from infant data, the initial parcellation scheme was developed using adult data, which may not appropriately fit the developing neonatal brain. Replicating these analyses using a neonatal derived parcellation scheme remains necessary. In addition, this work was completed using manually drawn brain masks for both training and testing data, which is a time consuming and expensive process. Future work is needed to adapt these methods to allow for unmasked data. For the 3DGAN-T2w method, this would mean improving memory efficiency to allow for the larger image inputs, and for the Kaplan-T2w method this would require initial unmasked image registration that can be used to automatically delineate the brain-skull boundary.

## 5 Conclusions

This work offers two innovative methods for synthetic image generation that can be used when one structural image modality is missing in neonatal MR analyses. Critically, both methods can be readily implemented using publicly available software, and output images can be successfully incorporated into existing MRI processing pipelines. Importantly, developing these methods in a neonatal population provides a means of avoiding subject loss due to inherent challenges associated with scanning this age group, and successful application of either approach will greatly assist future studies of brain-behavior relationships in the developing brain.

## Data and code availability statement

All data and code developed and/or used specifically for this study can be made available to qualified investigators by written request through the study authors under the guidance of a formal data sharing agreement between institutions that includes: 1) using the data only for research purposes and not attempting to identify any participant; 2) limiting analyses to those described in both institutions IRB-approved protocols; and 3) no redistribution of any shared data without a data sharing agreement.

## CRediT authorship contribution statement

**Sydney Kaplan:** Writing – original draft, Writing – review & editing, Methodology, Formal analysis, Visualization. **Anders Perrone:** Writing – original draft, Writing – review & editing, Methodology, Visualization. **Dimitrios Alexopoulos:** Conceptualization, Formal analysis. **Jeanette K. Kenley:** Conceptualization, Formal analysis. **Deanna M. Barch:** Funding acquisition, Investigation. **Claudia Buss:** Funding acquisition, Investigation. **Jed T. Elison:** Funding acquisition, Investigation. **Alice M. Graham:** Funding acquisition, Investigation. **Jeffrey J. Neil:** Conceptualization. **Thomas G. O'Connor:** Funding acquisition, Investigation. **Jerod M. Rasmussen:** Funding acquisition, Investigation. **Monica D. Rosenberg:** Funding acquisition, Investigation. **Cynthia E. Rogers:** Funding acquisition, Investigation. **Aristeidis Sotiras:** Conceptualization. **Damien A. Fair:** Writing – original draft, Funding acquisition, Investigation, Supervision. **Christopher D. Smyser:** Writing – original draft, Writing – review & editing, Funding acquisition, Investigation, Supervision.

## Declaration of Competing Interest

The authors do not report any competing interests.

## Appendix Supplementary materials

Image, application 1
