# Supplementary material for: Synthesizing pseudo-T2w images to recapture missing data in neonatal neuroimaging with applications in rs-fMRI
Source: Neuroimage. 2022 Jun;253:119091. doi: 10.1016/j.neuroimage.2022.119091 (PMC9127394; doi:10.1016/j.neuroimage.2022.119091)
Supplement: Supplementary file 1 [file mmc1.docx]

**Supplemental Information**

**3D-CycleGAN Additional Analyses**

The training framework for the full 3DCycleGAN is depicted in SI Fig. 1.


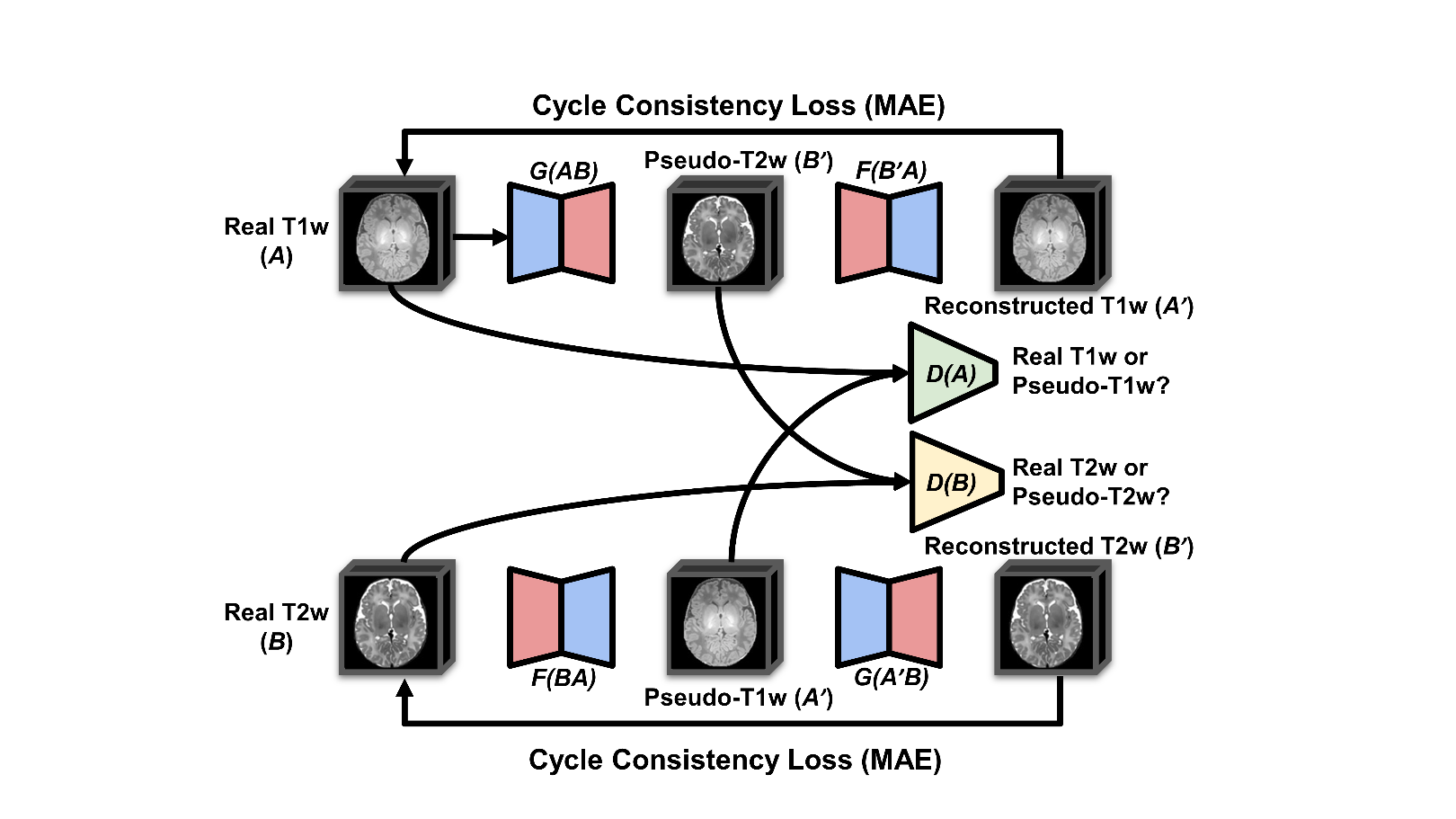


**Supplementary Figure 1: 3D-CycleGAN Training Framework.** The training procedure consists of multiple neural networks trained simultaneously with the goal of learning non-linear mappings *G:A🡪B* and *F:B🡪A*, where *A* is the domain of T1w images, *B* is the domain of T2w images, and *G* and *F* are U-Nets, termed “generators”, such that the distribution of images from *G(A)* is indistinguishable from the distribution of *B* and vice versa. Generator networks are trained via their associated adversarial discriminators, *D(B)* and *D(A),* both of which are convolutional neural network (CNN) classifiers that are trained to discriminate synthetic images from real images. *D(B)* is randomly given real or synthetic images from domain *B*. *D(B)* then updates *G* based on how easily it can identify a synthetic image thus encouraging it to translate A into outputs indistinguishable from domain *B*, and vice versa for *D(A)* and *F*. To further regularize the mappings, two cycle consistency losses were included to capture the intuition that translating from one domain to the other and back again should return the original image. The use of transitivity here creates a form of semi-supervised learning where the target is known even if the dataset is unpaired (Zhu et al., 2017).

The 3D-CycleGAN was trained multiple times using an incremental number of subjects (N) to determine the minimal N required for adequate out-of-sample image synthesis. To evaluate the performance of each model, we computed the mean squared error (MSE) between the pseudo-T2w and target T2w for each of the 20 test subjects at the end of each training epoch. We bootstrapped this approach collecting 10 random samples for each N training subjects. The bootstrapped mean and confidence interval (CI) of the MSE over 50 epochs at each N, as well as volumetric image slices from a representative test subject’s pseudo-T2w generated from each model are shown in SI Fig. 2. As N increases, the model stabilizes more quickly to a lower average MSE with a slightly more narrow CI. While the MSE plateaus to a minimum at a training sample size of N=70, image quality differences on qualitative inspection are almost imperceptible after N=50. Further research is needed to determine what effect psuedo-T2w images generated from models below this threshold have on segmentations and FC estimates.

**
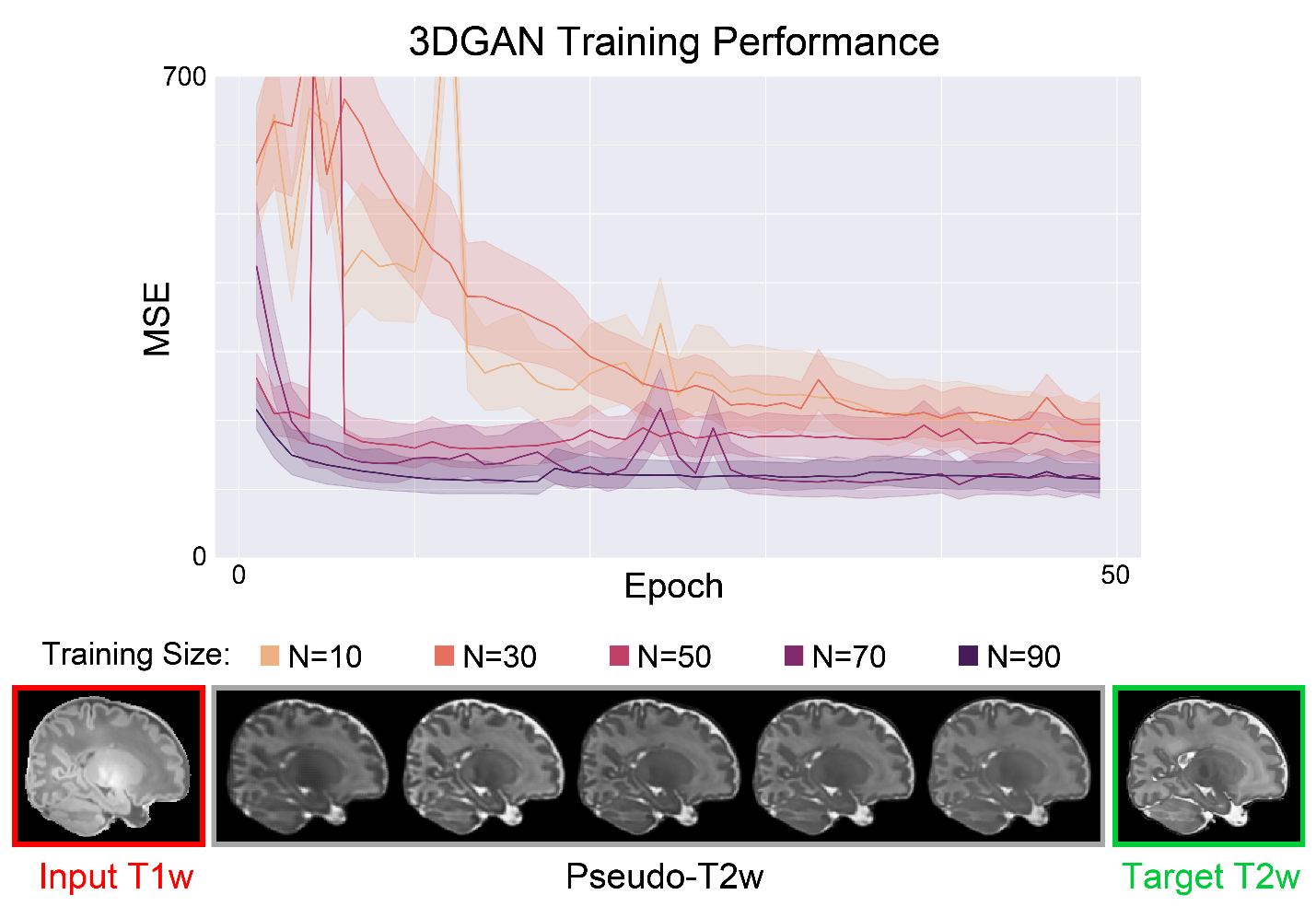
**

**Supplementary Figure 2: 3D-cycleGAN performance by training size.** The accuracy of pseudo-T2w images generated indicated by the MSE with the T2w during each epoch of training for various training set sizes. Representative pseudo-T2w images for each training set size show improved image quality compared to the target as N increases.

**Additional Comparisons**

Visual comparison of surfaces derived using pseudo-T2w and T2w images is shown in SI Fig. 3.


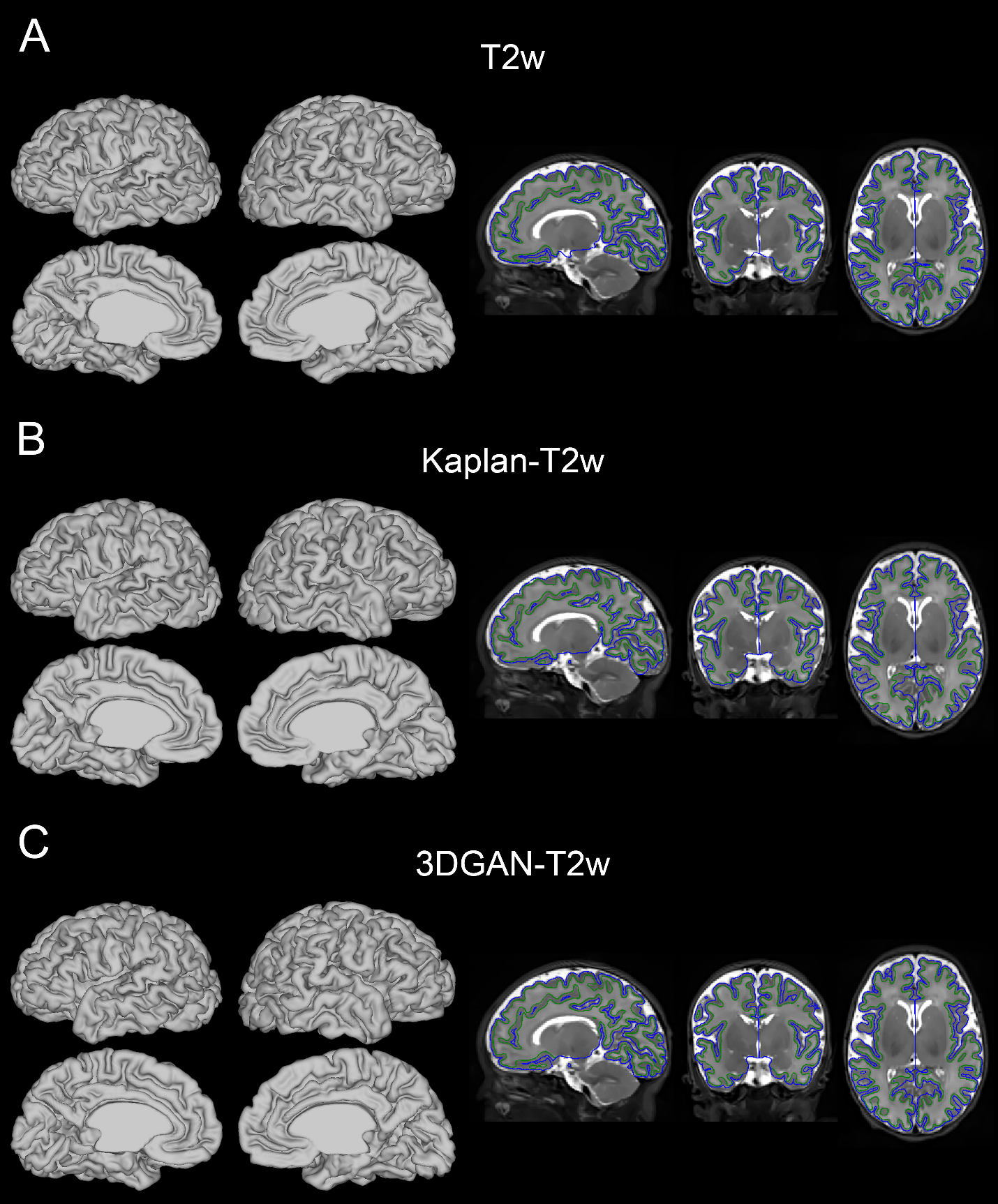


**Supplementary Figure 3: Surface comparison of pseudo-T2w images to T2w images.** Midthickness surfaces (left) as well as white and pial surface contours (right) derived from (A) T2w, (B) Kaplan-T2w, and (C) 3DGAN-T2w images.

To confirm T2w and pseudo-T2w images result in improved registration in comparison to using the T1w images, the registration analyses were replicated using only the T1w for each participant. This registration was computed using T1w atlases analogous to the T2w atlases to avoid bias due to the difference in sequence. The resultant MI for all subjects using each anatomical image registration target is depicted in SI Fig. 4. As demonstrated, the average MI across subjects was significantly higher for both the T2w and pseudo-T2w images in comparison to the T1w images (p<0.001) across both forms of registration.


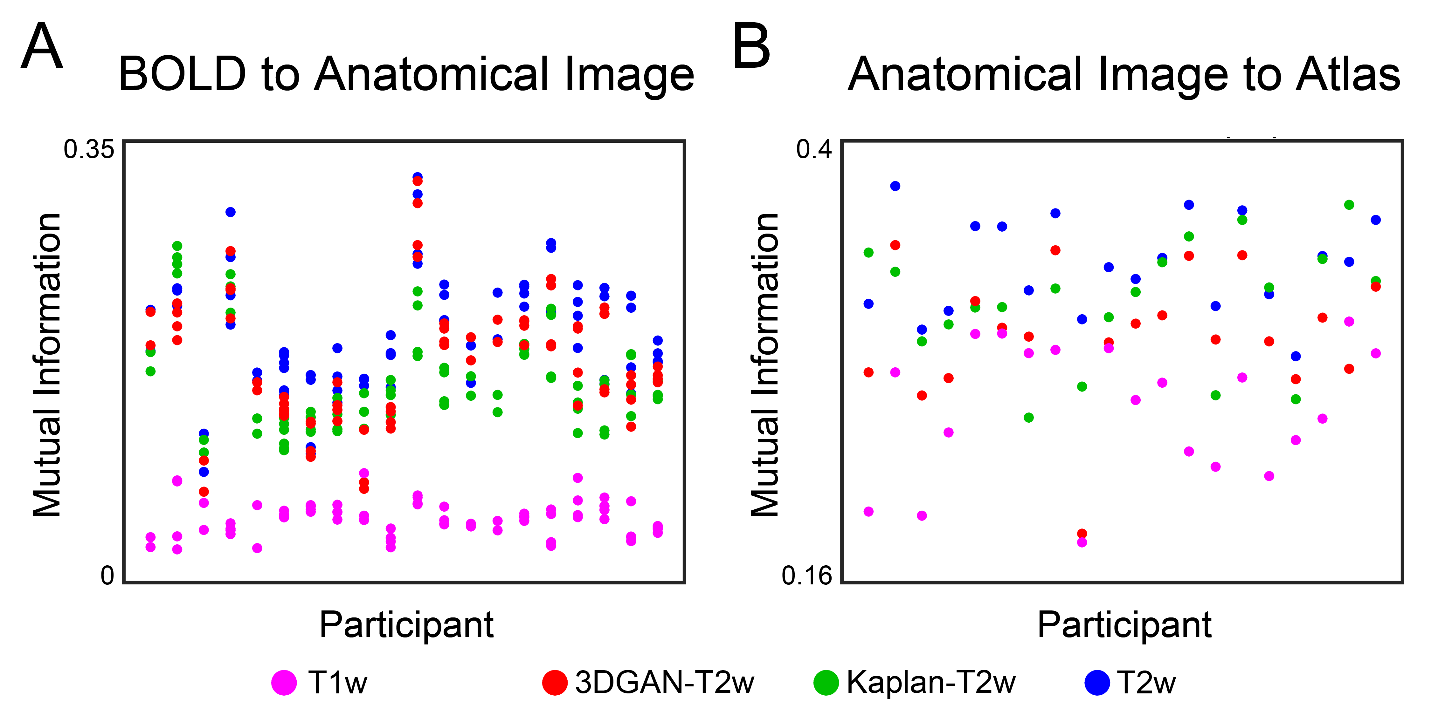


**Supplementary Figure 4: Pseudo-T2w and T2w image registration compared to T1w.** Registration quality for each participant measured as MI between (A) BOLD and anatomical images, as well as (B) registered anatomical and atlas images for each anatomical image type. Note the greater MI of T2w and pseudo-T2w images compared to the T1w images.

**ECHO Cohort Results**

All analyses described in the main text were replicated in an independent cohort (ECHO) using the same models that were trained on the eLABE dataset to generate pseudo-T2ws, the results of which are presented here. The mean of the absolute errors (MAE) for all ECHO subjects between T2w and pseudo-T2w images is presented in SI Fig. 5. Relative MAE across subjects was 22.7±3.4% for Kaplan-T2w and 22.9±1.6% for 3DGAN-T2w images. The slight increase in error relative to the eLABE study can be attributed to the difference in T2w sequence parameters that affect the T2w contrast properties between the training and testing data (eLABE and ECHO, respectively). Note, however, the anatomical similarity is retained.


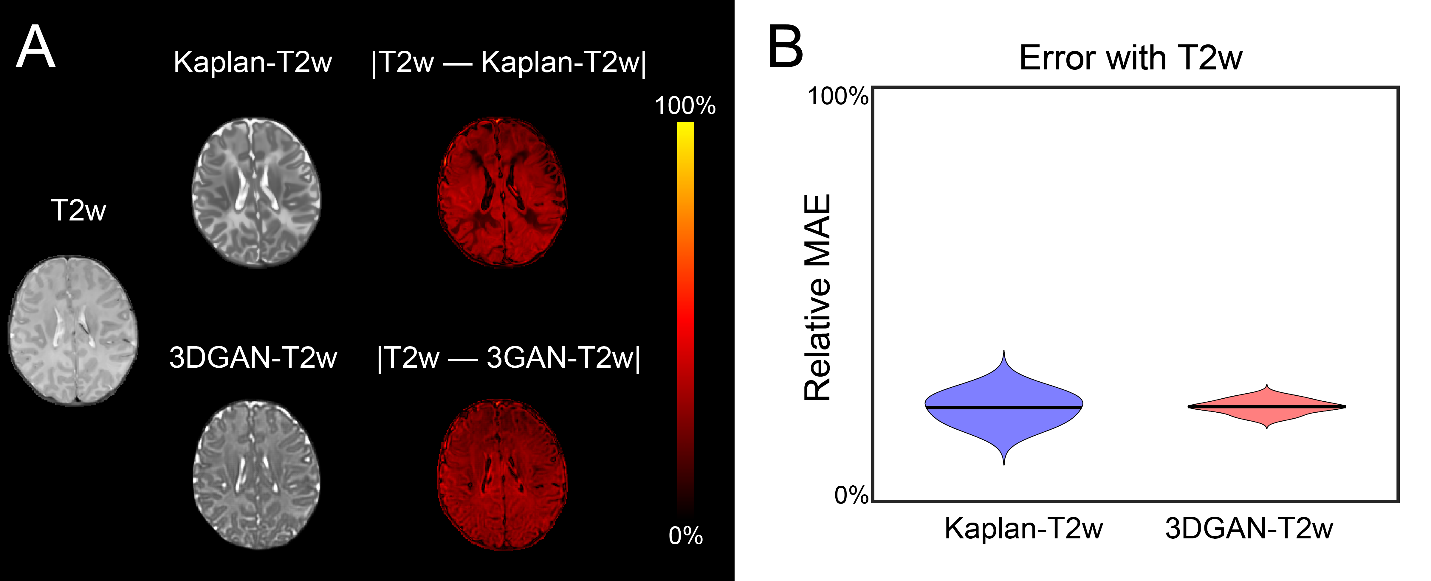


**Supplementary Figure 5: Error in pseudo-T2w images (ECHO subjects).** (A) Heatmap of absolute error between each pseudo-T2w image and the corresponding T2w image for a representative subject. Brighter values indicate larger error which are reflective of the difference in T2w sequence parameters that affect contrast between training and testing data. (B) Violin plot depicting the relative MAE with T2w images of all subjects for both peudo-T2w images (Kaplan-T2w 22.7±3.4%, 3DGAN-T2w 22.9±1.6%). Smaller values indicate less error.

SI Fig. 6 shows CNR for 3DGAN-T2w, Kaplan-T2w, and T2w images for all ECHO subjects. CNR is equivalent between T2w and Kaplan-T2w images (p=0.27) and larger in T2w than 3DGAN-T2w images (p=0.01).


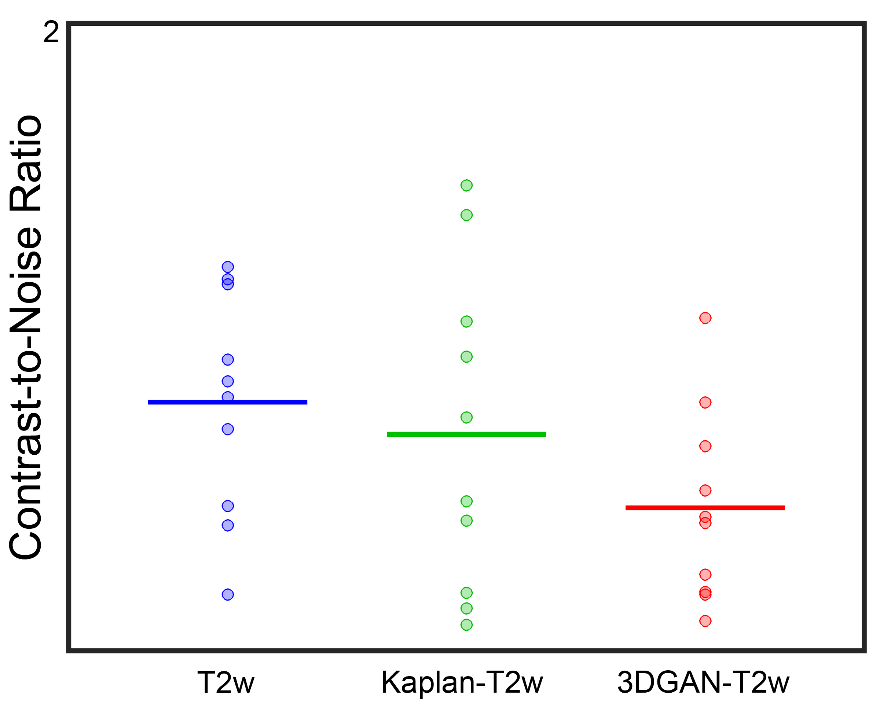


**Supplementary Figure 6: Contrast comparison of anatomical images (ECHO subjects).** CNR between gray and white matter of different anatomical image types. CNR distributions are equivalent between T2w (0.79±0.36) images and Kaplan-T2w (0.69±0.51), and there is substantial overlap with the 3DGAN-T2w images (0.46±0.31).

Registration quality of BOLD to anatomic data and anatomic data to the 711-2N Talairach atlas is shown in SI Fig. 7. Mean MI for BOLD to anatomic data was: 3DGAN-T2w 0.21±0.04, Kaplan-T2w 0.18±0.05, and T2w 0.25±0.06, where T2w registration was higher than both pseudo-T2w images for BOLD registration (p<0.001 for both Kaplan-T2w and 3DGAN-T2w). Mean MI for the mean anatomic data to the 711-2N Talairach atlas was: 3DGAN-T2w 0.24±0.03, Kaplan-T2w 0.32±0.04, and T2w 0.26±0.02, where T2w and 3DGAN-T2w images produce equivalent registrations (p=0.12), and Kaplan-T2w had higher anatomical registrations (p<0.001 for both T2w 3DGAN-T2w).


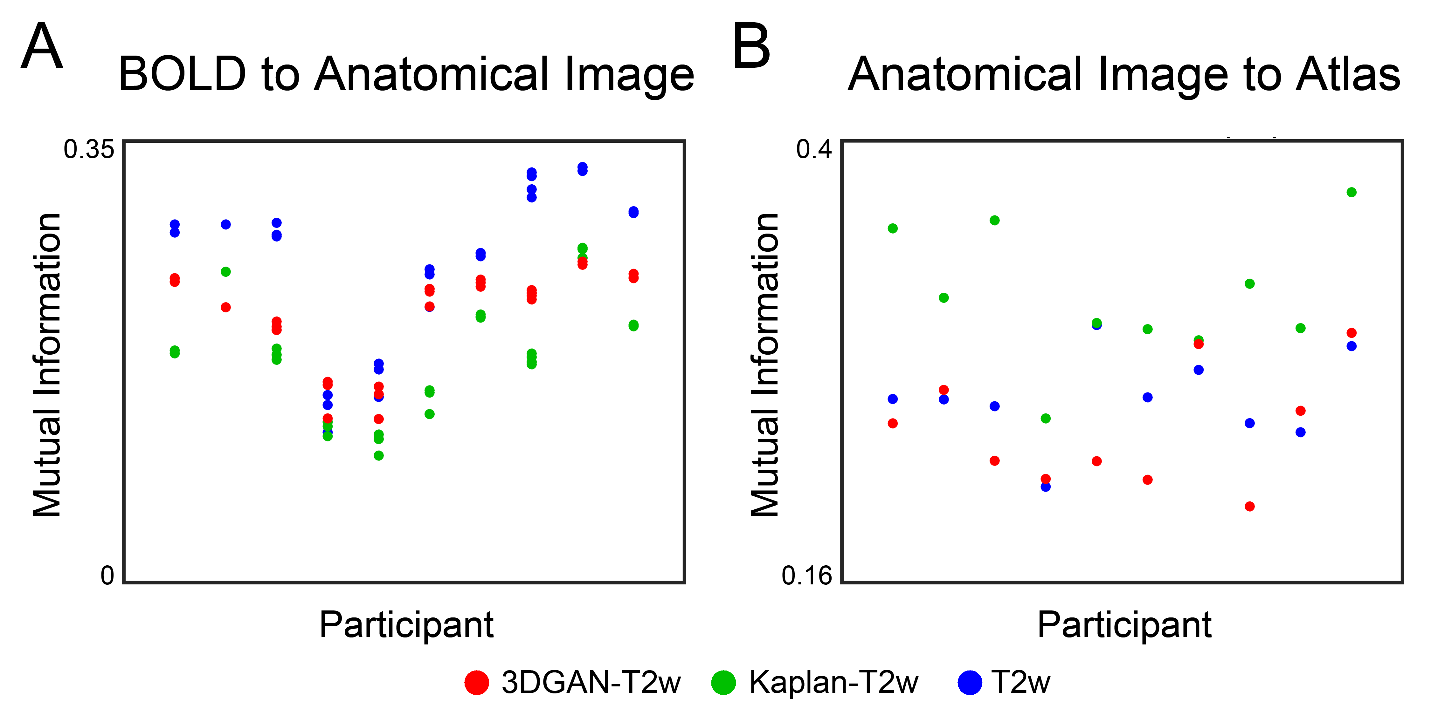


**Supplementary Figure 7: Registration accuracy of BOLD and anatomical images (ECHO subjects).** Mutual information (MI) for each participant between registered (A) BOLD and anatomical images, as well as registered (B) anatomical and atlas images for each anatomical image type.

Dconns of pseudo-T2w images with the T2w maps were highly correlated (R=0.95 for Kaplan-T2w and R=0.93 for3DGAN-T2w). Paired t-test differences between psuedo-T2w and T2w connectivity matrices are shown in SI Fig. 8, where differences are minimal (0.01% for both Kaplan-T2w and 3DGAN-T2w).


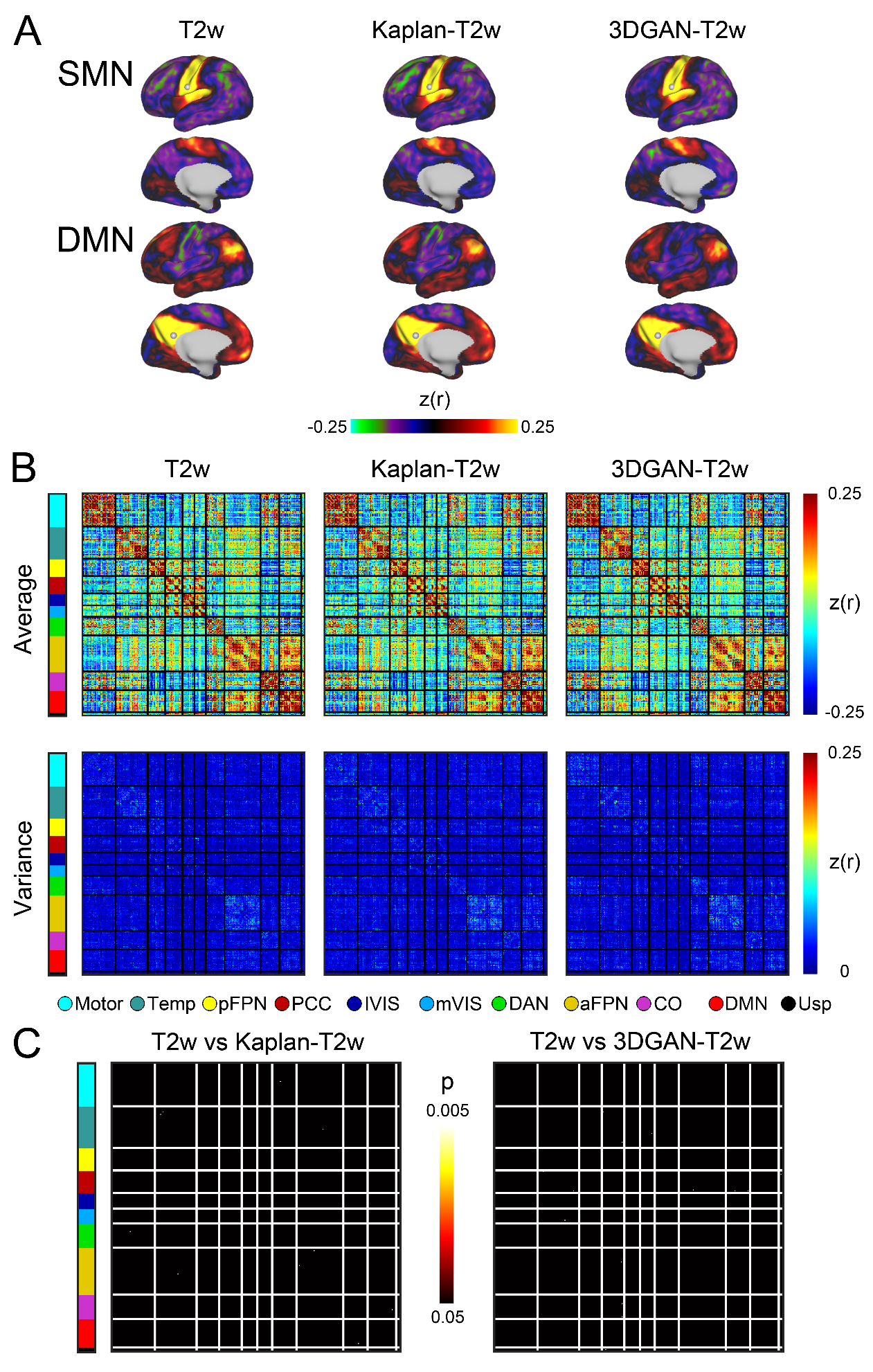


**Supplementary Figure 8: Pseudo-T2w use in functional connectivity (ECHO subjects).** (A) Average functional dense connectomes at select seeds for BOLD data pre-processed with T2w and psuedo-T2w images. Seedmaps from the somatomotor (top row) and default mode (bottom row) networks show similar connectivity patterns across the three anatomical image types. (B) Average functional connectivity matrices (top row) using cortical parcels and the variance of the connectivity matrices (bottom row) across participants for BOLD data pre-processed with T2w and pseudo-T2w images. (C) FC estimate differences between BOLD data pre-processed with T2w images and Kaplan-T2w or 3DGAN-T2w images. Brighter colors indicate more significant differences.
